# The relevance of nanotechnology, hepato-protective agents in reducing the toxicity and augmenting the bioavailability of isotretinoin

**DOI:** 10.1080/10717544.2020.1862365

**Published:** 2020-12-23

**Authors:** Khaled M. Hosny, Nabil A. Alhakamy, Khalid S. Al Nahyah

**Affiliations:** a Department of Pharmaceutics, Faculty of Pharmacy, King Abdulaziz University, Jeddah, Saudi Arabia; b Center of Excellence for Drug Research and Pharmaceutical Industries, King Abdulaziz University, Jeddah, Saudi Arabia

**Keywords:** Isotretinoin, acne, nanoemulsion, resveratrol, hepatoprotective, mixture design

## Abstract

Acne Vulgaris is one of the most common chronic inflammatory skin disorders that affect majority of teen-agers worldwide. Isotretinoin (ITT) is the drug of choice in the management of acne, but, it suffers from serious side-effects including hepatotoxicity, and some psychological disturbances following its oral intake. The objective of this study was to develop and optimize ITT loaded nanoemulsions (ITT-SNEDDS) and to incorporate resveratrol (RSV)in optimum formulation to decrease ITT side effects The ITT solubility was first tested in various essential oils, surfactants, and co-surfactants to select the essential nanoemulsion ingredients. Mixture design was applied to study the effect of independent variables and their interactions on the selected dependent responses. The developed ITT-SNEDDS were characterized for their globule size and *ex vivo* permeation. The optimized batch was further loaded with RSV and evaluated for in vitro and *ex vivo* permeation and for in vivo hepatotoxicity. The developed ITT-SNEDDS exhibited globule size below 300 nm, up to 272.27 ± 7.12 mcg/cm^2^.h and 61.27 ± 2.83% of steady-state flux (J_SS_) and permeability % respectively. Optimum formulation consisted of 0.15 g oil mixture, 0.6 g of surfactant (Labrasol), and 0.250 g co-surfactant (Transcutol). Permeability studies confirmed the enhanced permeation percentage of ITT (40.77 ± 1.18%), and RSV (29.94 ± 2.02%) from optimized formulation, with enhanced steady-state flux (J_SS_). In vivo studies demonstrated the superior hepatoprotective activity of optimized formulation compared to a different drug formulations and marketed product. Therefore, RVS loaded ITT-SNEDDS might be a successful strategy for acne management with improved action, and minimum side effects.

## 1. Introduction

Acne, the most frequent dermatological condition, is observed typically in adolescents, but it may affect individuals of all ages (Decker & Graber, 2012). The treatment effects can be assessed only after 2–3 months of use, and the therapy can be manipulated (reduced or maintained or increased) accordingly (Asai et al., 2016). Among several medications for dermatological application, isotretinoin (ITT) has been the most widely used drug. Chemically, ITT is (2Z, 4E, 6E, 8E)-3,7-Dimethyl-9-(2,6,6-trimethylcyclohex-1-enyl)nona-2,4,6,8-tetraenoic acid. The oral form of ITT (13-*cis*-retinoic acid) was initially approved by the USFDA (U.S. Food and Drug Administration) in 1982 to treat severe acne (Layton, 2009), and its application has been extended to reduce the development of sebum to significantly trim down the inflammatory lesions (Nelson et al., 2006). ITT has a unique importance in treating adult women with acne, as it is non-hormonal and non-antibiotic. ITT is effective because of the improved production of lipocalin through neutrophil gelatinase (anti-microbial protein). Over the decades, no other drugs or treatments have diminished the effectiveness of ITT. Nonetheless, despite these fascinating features, extremely low solubility and permissibility can limit the incorporation of ITT into a suitable vehicle and lead to poor patient compliance. Consequently, it is prudent to enhance the solubility and minimize the adverse effects using an appropriate carrier.

Stilbenes and natural phenolic compounds are found in various food sources, especially berries. One such compound is resveratrol (RSV), chemically 3,4,5-trihydroxystilbene, available in two different forms, *cis*-RSV and *trans*-RSV, with the *trans* form being a more stable compound (Baxter, 2008; Kala et al., 2012). RSV has been extensively researched because of its renowned biological activities. Pharmacologically, the compound has been shown to have anti-aging (De La Lastra & Villegas, 2007), anti-inflammatory (Baxter, 2008), cardio-protective (Das & Das, 2007), anti-oxidant (Hung et al., 2000), and anti-carcinogenic (Chen et al., 2015) effects. Additionally, RSV can decrease the levels of alanine aminotransferase (AST), aspartate aminotransferase (ALT), triglycerides, LDL-Cholesterol, and total cholesterol by regulating liver lipid metabolism (Peiyuan et al., 2017). RSV has been proven as an efficient anti-inflammatory and hepatosteatosis material in controlling the alcoholic liver disease. As Peiyuan et al. indicated, the hepatoprotective effect of RSV can be measured by regulating several pathways, such as apoptosis, oxidative stress, and swelling (Planas et al., 2012). Drug-mediated hepatotoxicity further confirmed the mechanism of RSV through the generation of free radicals and cytokines. RSV is significant in treating several liver diseases due to its antioxidant and anti-inflammatory properties.

The potential application of RSV as a therapeutic agent has been limited due to photosensitivity and partial intestinal absorption, thus decreasing bioavailability and causing extensive elimination and catabolism through ABC transporters (Karthikeyan et al., 2013; Vargas et al., 2016). Various formulations, including loading RSV into solid lipid nanoparticle, liposomes, and gelatin nanoparticles, have been applied to conquer these limitations (Teskač & Kristl, 2010; Coimbra et al., 2011; Abdelbary et al., 2017).

Topical route is a very well established pathway of drug administration that have the advantage of improved local therapeutic effect, convenience of use, and the possibility of stopping treatment if necessary (Satalkar et al., 2016). It also avoids drug metabolism in liver and hence maximizes the therapeutic benefit of drugs

Self-nano emulsifying delivery systems (SNEDDS), on dilution, produce nano-sized transparent systems called nanoemulsions (Khalid & El-Sawy, 2017; Hosny et al., 2019; Alshehri et al., 2020). These systems have been researched extensively, as they can offer higher stability and enhanced solubility, finally, improving the bioavailability of many insoluble or partially soluble therapeutic agents (Venkatesh & Mallesh, 2013; Wu et al., 2013). Nanoemulsions can improve the efficacy and functionality of several natural and chemical active moieties. Many therapeutic agents, like antioxidants, non-steroidal anti-inflammatory drugs, and lipids, were successfully formulated using nanoemulsions as novel carriers for topical application. Besides, nano-emulsion has also been used for the decontamination of radio nuclei and photodynamic therapy. Consequently, nanoemulsions demonstrate the enormous application in the area of dermatology (Aqil et al., 2016).

Topical SNEDDS have shown good properties compared to other topically applied drug delivery systems (Çinar, 2017; Alshehri et al., 2020). Such advantages include, being noninvasive, cost-effective, able to entrap a broad spectrum of hydrophilic and hydrophobic drugs, ability to protect several agents from hepatic metabolism and diminish their unwanted side effects. Moreover, SNEDDS’ components often acquire good anti-microbial and anti-viral characteristics which results in well preserved formulations with prolonged stability. SNEDDS are usually easily developed since they require no sophisticated procedures or high energy methods (Sarker, 2005; Hosny et al., 2020). Although SNEDDS resemble microemulsions (MEs) in many aspects, however, NEs are more kinetically stable and also have much lower globule size that allow them to enhance drug dissolution and permeation through skin ending up increasing the drug’s therapeutic efficiency (Pathania et al., 2018).

Numerous scientific works were carried out over the years on the application of essential oils in formulating nanoemulsions. As the nanoemulsion comprises translucent, unstable immiscible phases, the prepared system is prone to flocculation, sedimentation, and coalescence (de Campos et al., 2012). Accordingly, essential oils have been used in formulating the nanoemulsions to acquire high stability. Furthermore, the utilization of essential oils in formulating nanoemulsions is readily scalable, economic, and eco-friendly (Malhi et al., 2017).

Tea tree oil extracted from Melaleuca alternifolia has a unique application in the treatment of acne. Owing to improved bacteriostatic activity and minimizing lesions formation, tea tree oil has been used to treat seborrheic dermatitis and acne vulgaris (Wren & Stucki, 2003). Baobab oil (extracted from Digitata Adansonia L) is an anti-diabetic, hepatoprotective agent, and several unique properties, like nonirritating hydration, support its potential application in the cosmetic area, as it can soften the dry skin by enormously absorbing the nutrients (Naguib et al., 2017).

Nanotechnology is the concept of manipulating active agents to be in the size range from 1 to 1000 nm. It is considered as the magic bullet for solving different problems associated with many drugs (Shafiq et al., 2007; Ahmed et al., 2015; Safwat et al., 2017; Yehia et al., 2017). The potential nanotechnology strategy circumvented the physicochemical and pharmacokinetic constraints of RSV and ITT. Nevertheless, very few studies have addressed the critical *in vivo* results of developed systems. Accordingly, this research intended to optimize the nanoemulsion-based formulation of RSV and ITT using Mixture design to improve their bioavailability and reduce their toxicity. Animal models were used to assess the hepatotoxicity of the optimized formulation.

## 2. Materials and methods

### 2.1. Materials

ITT was purchased from Wuhan Senwayer Century Chemical Co., Ltd (China). Essential oils were obtained from Hayward, CA, USA. RSV, was procured from Water Import and Export Co., Ltd (China). Triglycerides, propylene glycol monolaurate, and glycerol monooleate, Labrasol, and transcutol were generously gifted by Gattefosse (France). All other reagents and chemicals used were of analytical grades.

### 2.2. Determination of ITT solubility

The solubility of ITT in various oils, surfactants, and co-surfactants was determined. An excess amount of ITT was dissolved in about 2 ml of each vehicle individually. The mixture was then shaken on a water bath (Model-1031, GFL Corporation, Germany) for 24 h, and the temperature was maintained at 37 ± 0.5° C. Once the equilibrium was attained, the resulting mixture was centrifuged (Sigma, 3K30 Centrifuge, UK) at 8° C for 1 h at 15,000 rpm. The formed supernatant was then diluted with a suitable quantity of methanol and analyzed quantitatively for ITT concentration using a UV-Visible spectrophotometer at 362 nm. The experiment was repeated three times, and the values were noted as mean ± standard deviation.

### 2.3. Pre-formulation studies

Nine nanoemulsion formulations were designed with different proportions of the selected oils, surfactant, and co-surfactant using different concentrations of ITT (20–60 mg) in the first formulation only, then ITT concentrations from 20 to 40 were used in formulations 2 and 3. After that, the drug concentration that yielded the lowest globule size in formulations from 1 to 3 was used in the rest of the formulations. The composition of the 9 formulations was presented in Table 1.

**Table 1.** Various formulation strategies to study the solubility of ITT.

No	Tea tree oil (g)	Labrasol (g)	Transcutol (g)	Baobab oil (g)
1	0.20	0.60	0.20	0.00
2	0.10	0.60	0.20	0.10
3	0.30	0.50	0.20	0.00
4	0.30	0.40	0.30	0.00
5	0.20	0.40	0.40	0.00
6	0.20	0.70	0.10	0.00
7	0.10	0.60	0.20	0.10
8	0.20	0.60	0.10	0.10
9	0.20	0.35	0.35	0.10

The SNEDDs were prepared as follows; ITT was added to the oil phase mixture; subsequently, a mixture of surfactant and co-surfactant was added. The formed mixture was further sonicated using Ultrasonic Processors (VCX 750, USA). Finally, the formed dispersion was added to distilled water drop by drop, and nanoemulsion was formed spontaneously on mixing at room temperatures (Ahmed et al., 2018).

The prepared SNEDDs were evaluated for their globule size, as follows; a specific quantity of each formulation was diluted with distilled water to form an aliquot of 20 ml, and samples were analyzed at 25° C using a dynamic light scattering technique in Zetatrac particle size analyzer (Miccrotrac Inc, PA, USA) (Salem et al., 2020).

Later on, 20 mg RSV were added to the best three formulations that gave the lowest globule size to detect in change in droplet size in case of using two active agents.

### 2.4. Mixture design for the development of ITT loaded SNEDDS

Statistical optimization of response surface methodology (R.S.M) has proven a proficient tool in optimizing the various parameters in a multifarious process to obtain the desired responses; thus, it has been applied to formulate ITT-SNEDDS. Among several models, the mixture design was selected to study the interaction of independent variables. Three factors were selected, namely, percentages of oil mixture [tea tree oil/baobab oil in ratio 1:1] (X_1_), surfactant [labrasol] (X_2_), and cosurfactant [transcutol] (X_3_; Table 2). Data from the previous pre-formulation studies were used to choose the levels of independent variables. All these components were used selectively in several ratios, with a total concentration equaling to 100%. The effect of all these selected variables on globule size (Y_1_), steady-state inflow (J_SS_) (Y_2_), and penetrability percentage (Y_3_) was studied (Shafiq et al., 2007). A total of 16 runs were projected, and the variables are summarized in Table 3. The correlations between the selected variables and responses were further analyzed by applying the regression equation using Statgraphics Centurion XV software-15.2.05 version (StatPoint, Inc., USA). SNEDDS formulations were optimized for minimum globule size, highest J_SS_, and maximum permeation percentage.

**Table 2.** Experimental mixture design (components levels and selected responses).

Component	Level		Response	Constraints
	Low	high		
oil mixture (%); (X_1_)	0.17	0.3	Mean globule size (Y_1_)	Minimum
surfactant (%); (X_2_)	0.4	0.6	steady state inflow (J_SS_) (Y_2_	Maimum
Co-surfactant (%); (X_3_)	0.1	0.3	penetrability percentage (Y_3_)	Maximum

**Table 3.** Mixture design for the development of ITT Loaded SNEDDS.

	Independent variables			Drug
Run	(A): Oil Mixture (Tea tree oil + Baobab oil)	(B): Surfactant (Labrasol)	(C): Co-surfactant (Transcutol)	Isotretinoin
1	0.3 g	0.4 g	0.3 g	20 mg
2	0.17 g	0.6 g	0.23 g	20 mg
3	0.21 g	0.6 g	0.18 g	20 mg
4	0.3 g	0.45 g	0.24 g	20 mg
5	0.22 g	0.53 g	0.23 g	20 mg
6	0.29 g	0.50 g	0.20 g	20 mg
7	0.21g	0.6 g	0.18 g	20 mg
8	0.3 g	0.6 g	0.1 g	20 mg
9	0.17 g	0.52 g	0.3 g	20 mg
10	0.22 g	0.48 g	0.29 g	20 mg
11	0.27 g	0.56 g	0.15 g	20 mg
12	0.22 g	0.53 g	0.23 g	20 mg
13	0.15 g	0.57 g	0.27 g	20 mg
14	0.15 g	0.57 g	0.27 g	20 mg
15	0.22 g	0.48 g	0.29 g	20 mg
16	0.29 g	0.50 g	0.20 g	20 mg

### 2.5. Preparation of ITT nanoemulsion

The different formulations of ITT nanoemulsion presented in Table 3 were prepared as previously mentioned in Section 2.3 (Ahmed et al., 2018).

### 2.6. Characterization of ITT nanoemulsion

Prepared ISN was characterized by various parameters to study the effect of selected variables.

#### 2.6.1. Measurement of globule size

The globule size of the developed ITT loaded SNEDDS was measured as previously described in Section 2.3.

#### 2.6.2. Ex vivo *permeation studies to evaluate J_SS_ and permeation percentage*

*Ex vivo* permeation study was conducted according to a previously reported method with modification (Kurakula et al., 2015). Wister albino male rats were collected from the Animal House for Pharmacy faculty, King Abdulaziz University. Hair of rats’ abdominal region was removed. Animals were sacrificed and certain the shaved abdominal skin (2 cm × 2 cm) was obtained, freed from the underlying tissues and finally used as a model permeation membrane. The Obtained skin was soaked in a phosphate buffer of pH 7.2 for one day before the commencement of the study. The permeation efficiency of SNEDDS was studied with a simple, low head dissolution testing instrument (PT-DT70, Siemensstr, Germany). The receptor compartment was filled with 900 ml of phosphate buffer with continuous stirring at 7 rpm. The temperature of the buffer solution was maintained at 37 ± 0.5 °C. Afterward, the skin segment was placed between the donor and receptor compartment so that the dermal side of the skin should be in close contact with the receptor compartment. The donor compartment contained volumes of SNEDDS equivalent to 20 mg ITT. At specific intervals, (1, 2, 3, 4, 5, 6, 8, 12 h) aliquots of 2 ml samples were withdrawn and analyzed using a UV-Visible spectrophotometer (Jenway 6705, USA). RSV and ITT were quantitatively measured at 362 nm and 299 nm, respectively. Finally, J_SS_ and permeation percentages were determined

### 2.7. Optimization of SNEDDS formulations

The obtained results from the experimental runs were further analyzed using ANOVA (Analysis of Variance) with multiple response optimization and RSM. Optimized formulation was selected based on the constraints of selected responses.

### 2.8. The rationale of mixture design

After the optimization of experimental runs, an optimized formulation was prepared according to the concentrations proposed by the software, and its various parameters were evaluated. The rationale for the selected experimental design was validated by comparing the experimental values with predicted values and calculating relative error (%). The following equation was used to calculate relative error (Khallaf et al., 2020).

(1) $$\text{Relative error}(\%)=\frac{\text{Predicted value}-\text{Practical value}}{\text{Predicted value}}\times 100$$

### 2.9. In vitro release studies

In vitro, drug release studies were performed to calculate the amount of ITT and RSV released for the optimized formulation according to a previously described method (Alvarez-Figueroa & Blanco-Mendez, 2001). Low head dissolution testing instrument (PT-DT70, Siemensstr, Germany) with dialysis cellulose membrane of 1.7 cm^2^ diffusion area (Cut off 14,000 Da pore size, Sigma-Aldrich, USA) was utilized. First, the dialysis membrane was soaked in phosphate buffer (pH 7.4) for 15 min at 25 ± 0.5° C. The receptor compartment was filled with phosphate buffer, and glass tubes covered with the dialysis membranes and filled with certain volume of the optimized formulation equivalent to 5 mg of each drug were placed in the medium with continuous rotations (75 rpm). The temperature was maintained at 37 ± 0.5° C throughout the experiment. Samples were withdrawn at various time intervals (1, 2, 3, 4, 6, 9, and 12 h), and ITT and RSV concentrations were analyzed at 362 and 299 nm, respectively.

### 2.10. *Ex vivo* permeation study of optimized formulation

The permeation study for optimum formulation was carried out as previously described in Section 2.6.2, however the permeation parameters for both ITT and RSV were calculated.

### 2.11. *In vivo* studies

Thirty-six male adult mice weighing 20.5 ± 1.5g were selected and adapted for seven days before the study. The animals were divided into six groups consisting of six animals in each group. Animal groups received the following treatments; group 1 received normal saline orally and served as negative control, while group 2 received oral aqueous dispersion of ITT. Moreover, group 3 received topical aqueous dispersion of ITT, group 4 received topical optimized ITT-SNEDDS formulation, group 5 received optimized topical ITT-RSV-SNEDDS and finally group 6 received the marketed topical cream. The dose of ITT in tested formulations was 5 mg/kg, and the treatment period was 14 days. The tested rats were maintained at temperature of 25 ± 0.5 °C and 30 − 70% of relative humidity and subjected to 12 h darkness and 12 h light cycle. At the end of the study period, animals were sacrificed with cervical dislocation, blood samples were instantly withdrawn and finally dysfunction of the liver was estimated by measuring AST, ALT, superoxide dismutase (SOD), malondialdehyde (MDA), and glutathione peroxidase (GSH-Px).

### 2.12. Statistical analysis

The *in vivo* results obtained were expressed as mean ± S.D and analyzed using Statistical Package for the Social Science, version 26 (IBM, SPSS). One-way ANOVA was followed by a post-hoc Tukey test to determine significant differences between quantitative variables. *p* Value <.05 was noted as statistically significant.

## 3. Results and discussion

### 3.1. ITT solubility studies

**Figure 1.**
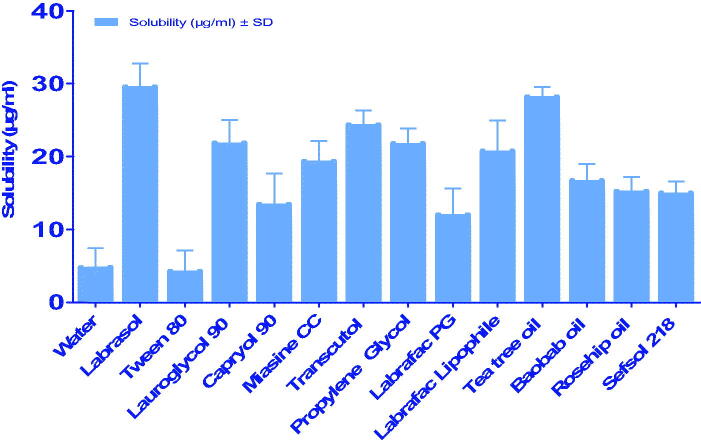
The solubility of ITT in assorted vehicles.

The solubility of ITT in assorted vehicles is depicted in Figure 1. The highest ITT solubility (29.54 ± 11.69 µg/ml) was found with Labrasol (a non-ionic-emulsifier), and comparable results were observed with other low soluble drugs, as specified by Patel et al. (Djekic & Primorac, 2008) In fact, labrasol could be considered one of the finest surfactants in increasing the solubility of hydrophobic drugs and it is worthy to mention that, Labrasol was frequently used for preparation of various topical micro and nanoemulsions (Djekic & Primorac, 2008; Moghimipour et al., 2012). Such character of Labrasol makes it the most productive element in improving the permeability and the most ideal candidate for SNEDDS production.

Also, ITT exhibited highest solubility with transcutol co-emulsifier (24.34 ± 14.28 µg/ml). Transcutol is known for its great miscibility in polar and non-polar solvents, as well as its competence to increase the absorption and degree of permeability (Osborne & Musakhanian, 2018; Vyas et al., 2010). Thus, it can serve as a carrier (co-surfactant) in formulating nanoemulsions. Essential oils also improved the solubility of ITT to a reasonable extent. Based on these results, surfactant-Labrasol, co-surfactant-Transcutol P, and essential oils (tea tree oil and baobab oil) were selected to formulate the nanoemulsion.

### 3.2. Preformulation studies

Globule sizes of all the prepared formulations were evaluated and data were presented in Table 4. First, the concentration of ITT to be used for further formulations was optimized. Formulation 1, having higher concentrations of ITT (60 mg, 40 mg), resulted in particle size of 315.03 ± 10.92 nm and 182.45 ± 25.81. However, ITT at 20 mg concentration formed the globules with lesser size (123.46 ± 5.85 nm). The identical phenomenon was observed with formulations 2 and 3. As a result, ITT of 20 mg concentration was used consistently to prepare the remaining formulations. Accordingly, decreased globule size was observed. Formulation 7 had the smallest globule size of 99.64 ± 4.69 nm. Formulation-9 had an elevated globule size of 299.66 ± 11.12 nm, which can be attributed to a higher concentration of Labrasol (0.35 g) and Transcutol (0.35 g).

**Table 4.** Composition of ITT in various formulations and their globule sizes.

No	ITT amount (mg)	Globule size (nm)
1	60	315.03 ± 10.92
	40	182.45 ± 25.81
	20	123.46 ± 5.85
2	40	369.89 ± 21.04
	20	213.98 ± 5.24
3	40	398.36 ± 29.66
	20	365.28 ± 5.42
4	20	212.51 ± 3.27
5	20	204.95 ± 6.44
6	20	131.34 ± 2.41
7	20	99.64 ± 4.69
8	20	198.85 ± 6.56
9	20	299.66 ± 11.12

### 3.3. Optimization of ITT loaded SNEDDS

Mixture design were used to analyze the effect of selected independent variables on minimum globule size (Y_1_), maximum J_SS_ (Y_2_), and high permeability percent (Y_3_) (Kurakula & Naveen, 2020). The observed responses of the 16 runs are given in Table 5. Globule size of all the trail batches was found to be in the range of 217.67–387 nm. J_SS_ and permeability percentages were estimated in the range of 98.54–272.27 mcg/cm^2^.h and 26.67%–61.27%, respectively. All obtained results were evaluated for individual responses. The effect of selected variables was further analyzed statistically using ANOVA and f_x_ model.

**Table 5.** Projected trail formulations and their observed responses according to Mixture design.

Run	Globule size*	J_SS_** mcg/cm^2^.h	% Permeated after 12 hours**
1	383.67 ± 66.58	113.67 ± 16.04	26.67 ± 7.40
2	251.13 ± 81.77	198.67 ± 5.03	54.46 ± 3.05
3	277.40 ± 61.04	200.36 ± 18.65	58.70 ± 4.11
4	373.33 ± 60.14	243.32 ± 4.60	52.75 ± 3.10
5	274.67 ± 56.80	164.79 ± 20.49	39.12 ± 3.55
6	304.57 ± 45.29	187.82 ± 0.30	42.57 ± 1.02
7	258.67 ± 33.28	249.07 ± 5.47	56.16 ± 2.99
8	284.20 ± 19.76	164.93 ± 4.31	45.97 ± 0.91
9	387.00 ± 10.15	98.54 ± 2.09	18.31 ± 0.74
10	309.27 ± 35.05	174.86 ± 10.66	42.53 ± 3.43
11	324.40 ± 41.22	195.77 ± 10.21	56.20 ± 0.58
12	330.47 ± 19.44	144.73 ± 28.68	36.50 ± 7.24
13	217.67 ± 8.39	272.27 ± 7.12	61.27 ± 2.83
14	222.67 ± 4.62	194.44 ± 2.87	50.21 ± 1.98
15	281.77 ± 90.97	154.92 ± 1.90	42.53 ± 3.43
16	286.73 ± 40.99	172.27 ± 16.40	40.82 ± 3.49

Based on the fit summary of responses (adjusted and predicted R^2^) and the sequential sum of squares (Type-I), different models were selected (Table 6). No models were misidentified with the highest order polynomial equations (Naveen et al., 2020). The accuracy of the model was further evaluated using the normal probability of studentized residuals. Externally studentized results were scattered around the straight line with a slight deviation. ANOVA was further used to quantitatively analyze the relationship between the selected independent variables and responses as follows;

**Table 6.** Fit summary for responses.

	Globule size (nm)		J_SS_ (mcg/cm2.h)		Permeation percentage (%)	
Source	Adjusted R²	Predicted R²	Adjusted R²	Predicted R²	Adjusted R²	Predicted R²
Linear	**0.8442**	**0.8091**	−0.0394	−0.4370	**0.8342**	**0.8014**
Quadratic	0.8094	0.7304	0.2233	−1.1593	0.7955	0.5349
Special Cubic	0.7924	0.6718	0.1612	−1.9809	0.7983	0.5079
Cubic	0.9753	−1.7131	**0.9924**	**0.8080**	0.9039	−5.2092

Bold values are significant at *p* < 0.01.

#### 3.3.1. Globule size (Y_1_):

The selected model was statistically significant, F = 41.64, *p* < .01%. Polynomial equations were further generated using multiple regression analysis. Both p values and polynomial equations were used to estimate the true effect of variables. *p* Values less than .0500 were considered statistically significant. Values greater than .1000 indicate the model terms are not significant. If there are many insignificant model terms (not counting those required to support hierarchy), the model reduction could have been required. The Lack of Fit F-value (0.97) was non-significant, with only a 1.25% chance that the Lack of Fit F-value this large could occur due to noise.

Furthermore, Adeq Precision measures the signal to noise ratio. In general, a ratio greater than 4 is desirable. The obtained ratio (17.910) indicated an adequate signal to navigate the design space (Kurakula et al., 2016). ANOVA results revealed the significant statistical relationship between the components and responses at a 95% confidence level.

Based on ANOVA results, Response (Y_1_) was affected significantly by the synergistic effect of liner mixture (*p* < .0001) (Guo et al., 2019; Kurakula & Naveen, 2020) with the highest magnitude (Table 7). The polynomial equation can be further applied to predict the response from any given concentration of independent variables. The equation was generated where lower concentrations of independent factors can contribute to the formation of globules with minimum size.

**Table 7.** ANOVA results in three responses.

Term	Responses					
	Globule size (nm)		J_SS_ (mcg/cm^2^.h)		Permeation percentage (%)	
	F-Value	*p* Value	F-Value	*p* Value	F-Value	*p* Value
Model	41.64	<.0001	219.94	<.0001	38.72	<.0001
Linear Mixture	41.64	<.0001	98.47	<.0001	38.72	<.0001
X_1_X_2_	–	–	466.41	<.0001	–	–
X_1_X_3_	–	–	663.70	<.0001	–	–
X_2_X_3_	–	–	253.56	<.0001	–	–
X_1_X_2_X_3_	–	–	431.04	<.0001	–	–
X_1_X_2_(X_1_−X_2_)	–	–	906.73	<.0001	–	–
X_1_X_3_(X_1_−X_3_)	–	–	540.90	<.0001	–	–
X_2_X_3_(X_2_−X_3_)	–	–	390.67	<.0001	–	–

Final Equations with Coded Factors

Globule size (Y_1_) = 481.52 ×_1_ + 110.81×_2_ + 260.11×_3_

**Figure 2.**
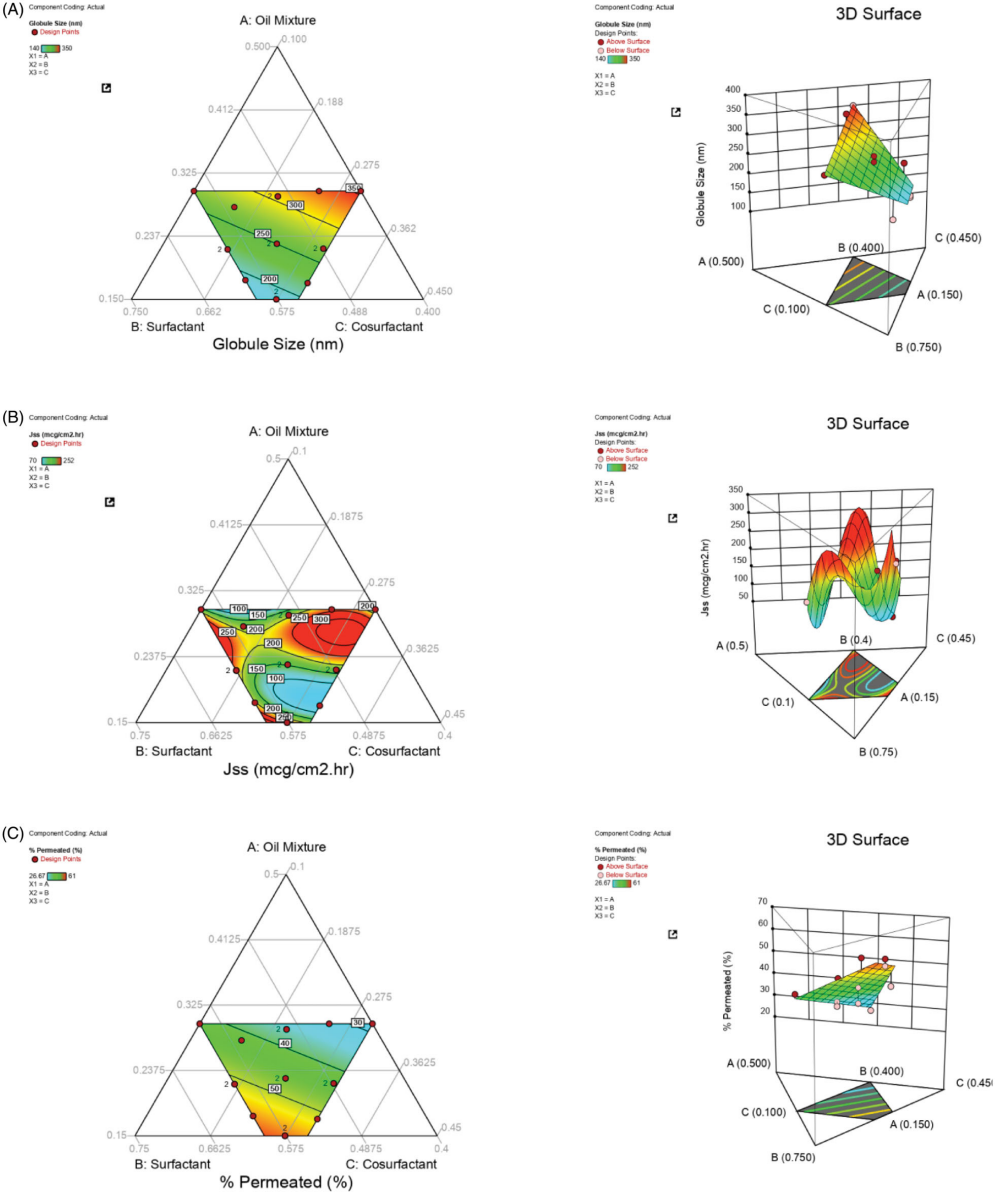
Contour and 3Dimensional response surface graphs for A) Globule size, B) J_SS_, and C) Percentage permeated.

Several studies have reported that increased oil levels can rise the globular size. This can be due to the coalescence of oil droplets and the expected decrease in the added surfactants or co-surfactants concentrations with the corresponding increase in oil percentage (Ahmed et al., 2018). Conversely, increasing the proportion of labrasol can reduce the globule size, due to its amphiphilic characters which enable it to decrease interfacial tension between oil droplets and surrounding aqueous media leading to smaller globule size (Naguib et al., 2017). RSM has been applied to analyze the effects of these selected factors. Contour plots and 3-dimensional graphs for Response I are shown in Figure 2. The mixture’s three components are depicted in the corners of the triangle, and the center portion illustrates the mixture.

#### 3.3.2. Steady-state inflow (J_ss_, Y_2_)

The model F value (219.94, *p* < .0001) and Lack of Fit F-value (0.7014) for Response (Y_2_) confirmed that the selected model is significant and the lack of fit is not significant, relative to the pure error. Adeq Precision (17.910) indicated an adequate signal to navigate the design space. Based on ANOVA results (Table 7), for the cubic model, X_1,_ X_3_, X_2_X_3_, X_1_X_2_X_3_, X_2_X_3_ (X_2_−X_3_) factors affected Response II antagonistically at a p value <.0001, and all remaining factors showed a synergistic effect. The generated polynomial equation shows that all these selected variables significantly affected J_SS_. Mixture design contour plots and 3D plots were similar to those for Y_1_. As discussed in the previous sections, higher concentrations of surfactants and oil mixture account for elevated and decreased J_SS_, respectively.

Final Equations with Coded Factors

J_SS_ (Y_2_) = − 12298.76 ×_1_ + 3087.44 ×_2_ − 494.73 ×_3_ + 17529.79 X_1_X + 25207.50 X_1_X_3_ − 4205.41 X_2_X_3_ − 21726.95 X_1_X_2_X_3_ +17752.15 X_1_X_2_ (X_1_−X_2_) + 13269.82 X_1_X_3_ (X_1_−X_3_) − 5101.24 X_2_X_3_ (X_2_−X_3_)

% permeated (Y_3_)

Adequate Precision for the linear model of Y_3_ was 17.19, which is greater than 4, suggesting that the model can navigate the design space. ANOVA results confirmed the significant effect of the linear mixture **(***p* < .0001). The selected three components are depicted in the corners of the triangle, and 3 D contour plots were drawn to study interaction effects. The fitted linear special regression equation was generated, as shown below.

Final Equations with Coded Factors

% Permeated = 5.02 ×_1_ + 69.94 ×_2_ + +45.46 ×_3_

The above results showed a higher significant effect of surfactant concentration compared to other variables. As the higher surfactant proportions can reduce the globule size, this can enhance the permeation percentage (Xia et al., 2013; Ahmed et al., 2014).

Labrasol was reported to inhibit the P-glycoprotein efflux transporters, thus could increase the permeation of several drugs. Labrasol might also cause stratum corneum fluidization through opening the tight junctions, which enhances permeability. All these reasons reinforce the potential application of surfactants and oils in formulating the nanoemulsions for topical delivery (Djekic & Primorac, 2008; Moghimipour et al., 2012). Transcutol was also reported to be able to penetrate and interact with the stratum corneum to modify the permeability of several therapeutic agents. Several studies have confirmed the enhanced solubility and permeability when using transcutol as a vehicle (Vyas et al., 2010).

**Figure 3.**
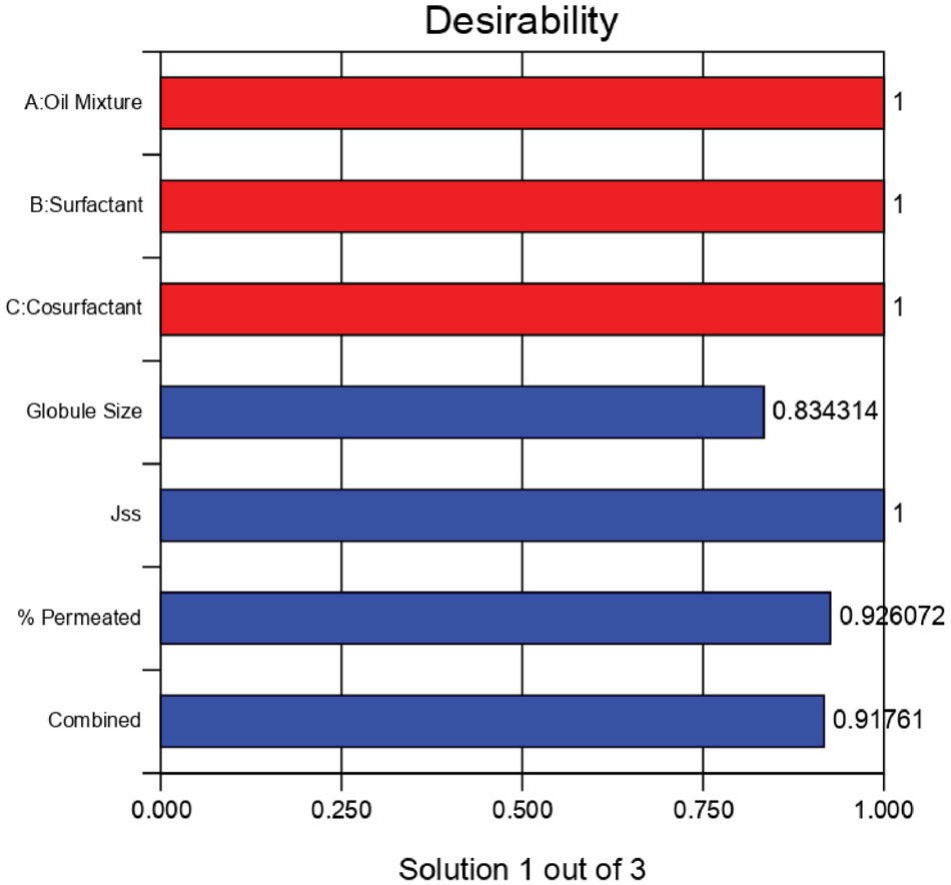
Bar chart for the desirability of the selected design.

The process was optimized by setting the goals for every response and simultaneously applying the Global desirability function (D). Based on these criteria, the desirability plot was generated with a D value of 0.918 (Figure 3). In conclusion, a formulation with 0.15 g oil mixture, 0.6 g of surfactant (labrasol), and 0.250 g co-surfactant (Transcutol) fulfilled the optimum formulation requirements. Using these variables can result in a formulation with a globule size of 174.79 nm, J_SS_-343.86, and permeation percentage of 59.45%. These predicted results were very close to the actual ones, which were 176.5 nm, 355.2 mcg/cm^2^.h) and 62.5% for globule size, J_ss_ and permeation percent, respectively.

### 3.4. Validation of experimental design

In the end, the experimental design was further validated to confirm accuracy. Optimized formulation was prepared with the given conditions and evaluated for globule size, J_SS_, and percentage permeation. Relative error was calculated by comparing the predicted and practical values, as shown in Table 8. Percentage bias was within the acceptable range (±5%), thus confirming the design’s preciseness. Optimum formulation loaded with RSV showed a globule size of 142.94 nm.

**Table 8.** Predicted vs. Experimental values for selected responses.

S. No	Parameter	Predicted values	Experimental Values	Percentage bias (%)	Adequate precision
1.	Globule size (nm)	174.79	176.25	−0.84	17.91
2.	J_SS_ (mcg/cm^2^.h)	343.86	343.12	0.21	56.410
3.	Permeation percentage (%)	59.45	61.15	−2.86	17.189

### 3.5. *In vitro* release/permeation study of optimized formulation loaded with RVS

The permeability percentage through cellulose membrane for RSV and ITT was found to be 42.52 ± 2.02% and 58.66 ± 0.77% after 12 h (Table 9). J_SS_ values further supported this finding. ITT had more J_SS_ values, resulting in higher permeation. In general, RSV has a slow permeation, but it can exhibit and retain antioxidant and anti-inflammatory effects on the skin.

**Table 9.** *In vitro* permeation and J_SS_ result of optimized formulation loaded with RSV.

Formulation	Item	J_SS_ mcg/cm^2^.h	% Permeated after 12 hours
RVS-ITT-SNEDDS	RVS	201.82 ± 9.97	42.52 ± 2.02
	ITT	221.56 ± 3.61	58.66 ± 0.77

### 3.6. *Ex vivo* permeation study of optimized formulation loaded with RVS

**Figure 4.**
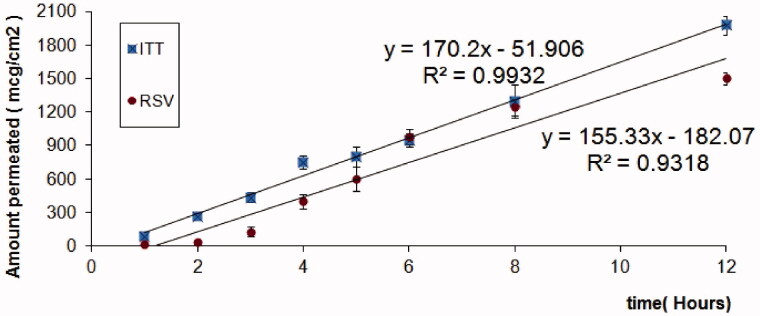
*Ex vivo* permeation diagram of ITT and RSV from optimized RVS-ITT-SNEDDS formulation.

*Ex vivo* studies were conducted using simple, low head dissolution apparatus to establish the effect of ITT-RSV formulation. The permeation was studied using rat full-thickness skin, and the results are summarized in Table 10. Increased permeation percentage of ITT (60.77 ± 1.18%) from the optimized nanoemulsion formulation was observed. This can be credited to the synergistic action of labrasol and transcutol to fluidize the cell membranes and inhibit P-glycoprotein efflux. Another rationale is the high permeation of nanoemulsion drug delivery systems. Nanoemulsion can act like a drug reservoir in which the loaded drug is released into the outer phase and then into the skin. Figure 4 illustrated the *ex vivo* permeation diagram of ITT and RSV from optimized RVS-ITT-SNEDDS formulation.

**Table 10.** *Ex vivo* permeation and J_SS_ results of optimized formulation loaded with RSV.

Formulation	Item	J_SS_ (mcg/cm^2^.h)	% Permeated after 12 hours
RVS-ITT-SNEDDS	RVS	155.35 ± 30.63	29.94 ± 2.02
	ITT	170.02 ± 0.34	40.77 ± 1.18

### 3.7. *In vivo* studies

Despite several notable advancements, hepatic safety and hepatic cell regeneration products are inadequate for treating several liver disorders. Numerous herbal extract formulations were also used in this approach (Gupta et al., 2010; Anantha et al., 2012). RSV is a natural flavonoid with exceptional antioxidant properties (Rubio-Ruiz et al., 2019; Yu et al., 2019; Daye et al., 2020). Significant alteration in hepato specific liquid peroxidation and oxidative stress was observed with ITT. A similar result was observed in a few previous studies (Vieira et al., 2012). Table 11 shows the elevation of ALT and AST levels observed with formulations containing ITT alone.

**Table 11.** The effect of various formulation ingredients on hepatoprotective activity in comparison to marketed formulation.

	AST		ALT		MDA		SOD		GSH	
Groups	AVG	STD	AVG	STD	AVG	STD	AVG	STD	AVG	STD
G1 [Conrol Normal saline]	18	1.8	23	1.5	0.611	0.024	421.8	2.8	677.1	1.8
G2 [Distilled water + Isotretinoin(Oral)]	165	19.3	80.6	2.6	1.14	0.025	307.3	3.5	323.1	1.5
G3 [Distilled water + Isotretinoin (Topical)]	65	8.1	45.5	1.8	0.82	0.018	326.1	1.9	473.5	1.9
G4 [Formula + Isotretinoin]	92.5	6.3	65.3	4.2	0.92	0.028	349.3	2.5	383.3	2.4
G5 [Formula + Isotretinoin + Resveratrol]	20.16	2.5	24.1	1.8	0.645	0.018	407.8	3.1	652.8	2.8
G6 [Marketed Isotretinoin cream]	63.1	5.2	50.8	1.1	0.88	0.021	348.8	3.8	421.16	2.1

Research by Vieira et al. confirmed the elevated triglyceride levels beyond the normal range in 11% of patients, increased AST in 8.36%, and increased ALT levels in 7.3% of patients. Consistent with these results, a significant rise in MDA levels, lipid peroxidation, and reduction in the antioxidant enzymes was supported by a significant decrease in the SOD activity and GSH-Px (Supplementary Figure 1).

The pretreatment with a formulation containing ITT + RSV for 14 days significantly increased the antioxidant property and reduced the lipid peroxidation, which reduced overall oxidative stress. All changes obtained with RSV formulation (Group 5) were more beneficial in ameliorating hepatotoxicity compared to other groups. Additionally, these two groups differed significantly from a marketed product (Group 6). No significant difference (*p* > .05) emerged between RSV-ITT SNEDDS formulation and the control formula for all tests. To conclude, the advanced formulation was developed relative to the marketed product in terms of hepatoprotective efficacy.

## 4. Conclusion

In this study, topical nanoemulsion was prepared to study the relevance of hepatoprotective agents, and utilization of nanotechnology in enhancing hepatoprotective activity. Based on solubility studies, surfactant-Labrasol, co-surfactant-Transcutol P, and essential oils (tea tree oil, and baobab oil) were selected to formulate the nanoemulsion. A low concentration of ITT favored the formation of globules with smaller particle sizes. Mixture design was successfully applied to optimize the concentrations of various parameters according to the desirability approach. Formulation with 0.15 g of oil mixture, 0.6 g of surfactant (labrasol), and 0.250 g of co-surfactant (Transcutol) achieved the optimum formulation. The range of relative error was acceptable (<5%), confirming the preciseness of the design. *In vitro* and *ex vivo* permeation studies confirmed the enhanced percentage of permeation due to the presence of labrasol and transcutol in the formulation. *In vivo* studies further supported these results. The hepatoprotective activity of the prepared formulation was superior compared to formulations containing individual drugs and commercially marketed products. Accordingly; RSV-ITT-SNEDDS can be considered a promising nano platform for using ITT in acne management with minimum side effects.

## Supplementary Material

Supplemental Material
