# Supplementary material for: The relevance of nanotechnology, hepato-protective agents in reducing the toxicity and augmenting the bioavailability of isotretinoin
Source: Drug Deliv. 2020 Dec 23;28(1):115–25. doi: 10.1080/10717544.2020.1862365 (PMC7758053; doi:10.1080/10717544.2020.1862365)
Supplement: Supplemental Material [file IDRD_A_1862365_SM6800.zip › Certification_For_language_editing.pdf]

SERVICESCAPE

Editing Certification

27 Congress Street, Suite 510
Salem, MA 01970
United States
(978) 594-0590

Invoice Number: 410888
Certification Date: 12/16/2020

To whom it may concern:

This letter shall serve as an official certification of professional editing and proofreading services. The document detailed below was edited by an experienced and well-qualified English editor.

Title:	Isotret.-Resver. nanoem
Author:	Khalid Omar
Editor:	Gabriela Dye
Language:	English

This document is certified to have been edited for proper language, style, punctuation, spelling, and grammar. Information regarding the editor's qualifications can be found at https://www.servicescape.com/editors/acadconsult.

The purpose of this certification is to declare the correctness of the edited document only. ServiceScape does not guarantee that the document is genuine or that the statements contained in the document are true. Furthermore, ServiceScape Incorporated assumes no liability for the way in which the edited document is used by the client or any third party, including end users of the edited document. ServiceScape Incorporated’s limitation of liability applies to this certification.

Please contact us if you have any questions.

Best Regards,

David Costello

David Costello
CEO and Administrator
ServiceScape Incorporated
info@servicescape.com
https://www.servicescape.com
