# Supplementary material for: The relevance of nanotechnology, hepato-protective agents in reducing the toxicity and augmenting the bioavailability of isotretinoin
Source: Drug Deliv. 2020 Dec 23;28(1):115–25. doi: 10.1080/10717544.2020.1862365 (PMC7758053; doi:10.1080/10717544.2020.1862365)
Supplement: Supplemental Material [file IDRD_A_1862365_SM6800.zip › Predicted_versus_actual_plots.doc]

**Response 1: Globule Size**

**Purterbation plot and Predicted versus Actual plot for globule size**

**Response 2: Jss**

**Purterbation plot and Predicted versus Actual plot for Jss**

**Response 3: % Permeated**

**Purterbation plot and Predicted versus Actual plot for % permeated**
